# Oxidative insults disrupt OPA1-mediated mitochondrial dynamics in cultured mammalian cells

**DOI:** 10.1080/13510002.2018.1492766

**Published:** 2018-07-01

**Authors:** Iraselia Garcia, Wendy Innis-Whitehouse, Alma Lopez, Megan Keniry, Robert Gilkerson

**Affiliations:** a Departments of Biology, The University of Texas Rio Grande Valley, Edinburg, TX, USA; b Biomedical Sciences, The University of Texas Rio Grande Valley, Edinburg, TX, USA; c Clinical Laboratory Sciences, The University of Texas Rio Grande Valley, Edinburg, TX, USA

**Keywords:** Transmembrane potential, oxidative stress, H_2_O_2_, OPA1, OMA1, fusion

## Abstract

**Objective:** To explore the impact of oxidative insults on mitochondrial dynamics. In mammalian cells, oxidative insults activate stress response pathways including inflammation, cytokine secretion, and apoptosis. Intriguingly, mitochondria are emerging as a sensitive network that may function as an early indicator of subsequent cellular stress responses. Mitochondria form a dynamic network, balancing fusion, mediated by optic atrophy-1 (OPA1), and fission events, mediated by dynamin-related protein-1 (DRP1), to maintain homeostasis.

**Methods:** Here, we examine the impact of oxidative insults on mitochondrial dynamics in 143B osteosarcoma and H9c2 cardiomyoblast cell lines via confocal microscopy, flow cytometry, and protein-based analyses.

**Results:** When challenged with hydrogen peroxide (H_2_O_2_), a ROS donor, both cell lines display fragmentation of the mitochondrial network and loss of fusion-active OPA1 isoforms, indicating that OPA1-mediated mitochondrial fusion is disrupted by oxidative damage in mammalian cells. Consistent with this, cells lacking OMA1, a key protease responsible for cleavage of OPA1, are protected against OPA1 cleavage and mitochondrial fragmentation in response to H_2_O_2_ challenge.

**Discussion:** Taken together, these findings indicate that oxidative insults damage OPA1-mediated mitochondrial dynamics in mammalian cells via activation of OMA1, consistent with an emerging role for mitochondrial dynamics as an early indicator of cellular stress signaling.

**Abbreviations**: Δψ_m_: transmembrane potential; ROS: reactive oxygen species; H_2_O_2_: hydrogen peroxide; OPA1: optic atrophy-1; MFN1: mitofusin1; DRP1: dynamin-related protein 1; DMEM: Dulbecco’s Modified Eagle’s Medium; PBS: phosphate buffer saline; TOM20: translocase of the outer mitochondrial membrane-20; DAPI: diaminophenylindole; TMRE: tetramethylrhodamine ethyl ester; TBST: Tris-Buffered Saline Tween-20; MEF: mouse embryonic fibroblast.

## 1. Introduction

Oxidative insults activate critical cellular stress response pathways, with severe outcomes including inflammation, proinflammatory cytokine secretion, and apoptosis. Oxidative stressors such as H_2_O_2_ cause apoptosis via activation of apoptosis-inducing factor (AIF) and caspase-3 [1,2], resulting in decreased cell viability [3]. H_2_O_2_ also engages inflammatory signaling, activating the inflammasome via induction of NLRP3 and subsequent secretion of proinflammatory cytokines including IL-1Β [4,5]. As such, oxidatively induced inflammation and apoptosis is a key mechanism in the pathogenesis of prevalent diseases including diabetes and cardiovascular disease [6]. Strikingly, the GTPase factors that direct mitochondrial fission/fusion dynamics play mechanistic roles in inflammatory and apoptotic signaling. OPA1 mediates fusion of the mitochondrial inner membrane [7] under control of the OMA1 metalloprotease [8,9], while DRP1 drives the opposing process of mitochondrial fission by actin-mediated recruitment to the outer mitochondrial membrane, followed by constriction and division of mitochondria [10,11]. Loss of either OPA1 or DRP1 severely disrupts mitochondrial dynamics, but also activates inflammatory signaling [12,13] via the NLRP3 inflammasome [4,6]. Mitochondrial dynamics, mediated by OPA1 and DRP1, thus maintain mitochondria as a highly sensitive cellular stress response network. To explore the impact of oxidative insults on mitochondrial dynamics as a general mechanism, we employ two cell lines, H9c2 cardiomyoblasts and 143B osteosarcomas, with very different origins and metabolic settings.

OPA1 and DRP1, along with other interacting fusion and fission factors, work cooperatively to maintain mitochondria as a highly responsive, dynamic organellar network with a high degree of interconnection. In response to stresses such as loss of mitochondrial transmembrane potential (Δψ_m_), however, the network collapses to a fragmented state, existing as a population of spherical organelles [14]. Mitochondrial fission/fusion balance thus requires coordination of multiple interacting factors. Outer membrane fusion is accomplished by mitofusin 1 (MFN1) and mitofusin 2 (MFN2) [15], while fusion of the inner membrane is mediated by OPA1. OPA1 is expressed in multiple isoforms, in which long (L-OPA1) isoforms mediate fusion of the inner membrane [7,16] and maintain inner membrane structure [17]. Conversely, mitochondrial fission, which produces a collection of disconnected spherical organelles, is accomplished by actin-mediated recruitment of DRP1 to the mitochondria [10], where it is bound by Fis1, MFF1, MiD49, and MiD51 [11,18,19], promoting formation of a DRP1 multimeric ‘collar’ for membrane scission [20,21] with Dyn2 [22]. Fission and fusion pathways directly interact: short OPA1 (S-OPA1) isoforms can activate mitochondrial fission [23], while DRP1 stabilizes L-OPA1 isoforms [24,25]. These dynamics have a hand-in-hand relationship with bioenergetic function: L-OPA isoforms are fusion-active, while loss of Δψ_m_ causes cleavage to fusion-inactive S-OPA1 [7], mediated by OMA1 [8,9]. Decreased Δψ_m_ also activates fission [26] via DRP1 dephosphorylation [27]. It is unclear, however, how oxidative insults affect this highly sensitive, dynamic organellar network. Here, we explore the impact of oxidative insults on mitochondrial fission/fusion dynamics as an early indicator of cellular stress.

## 2. Materials and methods

### 2.1. Cell culture

Human 143B osteosarcoma cells FLP6a39.2 (gift of Eric Schon, Columbia University, New York, NY, USA) and H9c2 cardiomyoblast (ATCC, Manassas, VA, USA) were grown in Dulbecco’s Modified Eagle’s Medium (DMEM) with 10% fetal bovine serum supplemented with 50 µg/mL uridine in 5% CO_2_ at 37°C. Cells were treated with 200 or 400 µM H_2_O_2_ for 1 h.

### 2.2. Fluorescence microscopy

Coverslips were fixed in 4% paraformaldehyde overnight at 4°C and blocked with 10% Normal Goat Serum (NGS) followed by anti-TOM20 antibody (1:100 dilution, Santa Cruz Biotechnology, Dallas, TX, USA) or DLP1 (1:500 dilution, BD Transduction 611112, San Jose, CA, USA) and AlexaFluor 488 goat anti-rabbit or goat anti-mouse antibody (1:100 dilution, Invitrogen Molecular Probes, Eugene, OR, USA). Slides were viewed on a Fluoview (FV10i) Olympus Confocal Microscope (Olympus America Inc., Melville, NY, USA) with a 60× UPLSAP60xW objective with 1.0 aperture and 3× optical zoom at room temperature.

### 2.3. Flow cytometry

Cells (∼10^6^ cell/dish) were treated with H_2_O_2_ for 40 min., incubated with 100 nM Tetramethylrhodamine, Ethyl Ester, Perchlorate (TMRE) (Invitrogen Molecular Probes, Eugene, OR) without or with H_2_O_2_ for 20 min and analyzed on a BD Biosciences Facscalibur (BD Biosciences, San Jose, CA, USA).

### 2.4. Western blotting

Cells were lysed in ice-cold Laemmli buffer containing 2-mercaptoethanol, run on a 6% polyacrylamide gel, and transferred to PVDF (Bio-Rad, Hercules, CA, USA). Membranes were incubated with primary antibodies overnight at 4°C, then incubated with secondary antibody, developed using WestDura (ThermoFisher, Waltham, MA, USA), and scanned using Gel Doc™ XR+ Gel Documentation System (Bio-Rad, Hercules, CA, USA). Antibodies: OPA1, 1:500 dilution (BD Transduction 612606, San Jose, CA, USA), DLP1, 1:1000 dilution (BD Transduction 611112, San Jose, CA, USA), anti-α tubulin, 1:1000 dilution (Sigma T6074, St. Louis, MO, USA) and goat anti-mouse poly-HRP secondary antibody, 1:3000 dilution (ThermoFisher 32230, Waltham, MA, USA).

### 2.5. Quantitative RT–PCR

For qRT-PCR, cells were grown in 100 mm dishes and treated with H_2_O_2_ for 1 h. Total RNA was prepared using the Qiagen RNeasy kit to generate cDNA using Superscript Reverse Transcriptase II (Invitrogen, Carlsbad, CA, USA). Samples were analyzed using SYBR green and the Eco Illumina Real-time system (Illumina, San Diego, CA, USA). Expression levels were normalized to actin. The oligonucleotide sequences used were: DRP1 forward primer ATGGCAACATCAGAGGCACT, DRP1 reverse primer TGGAATAACCCTTCCCATCA.

### 2.6. Quantitation of morphology and statistical analysis

**Figure 1.**
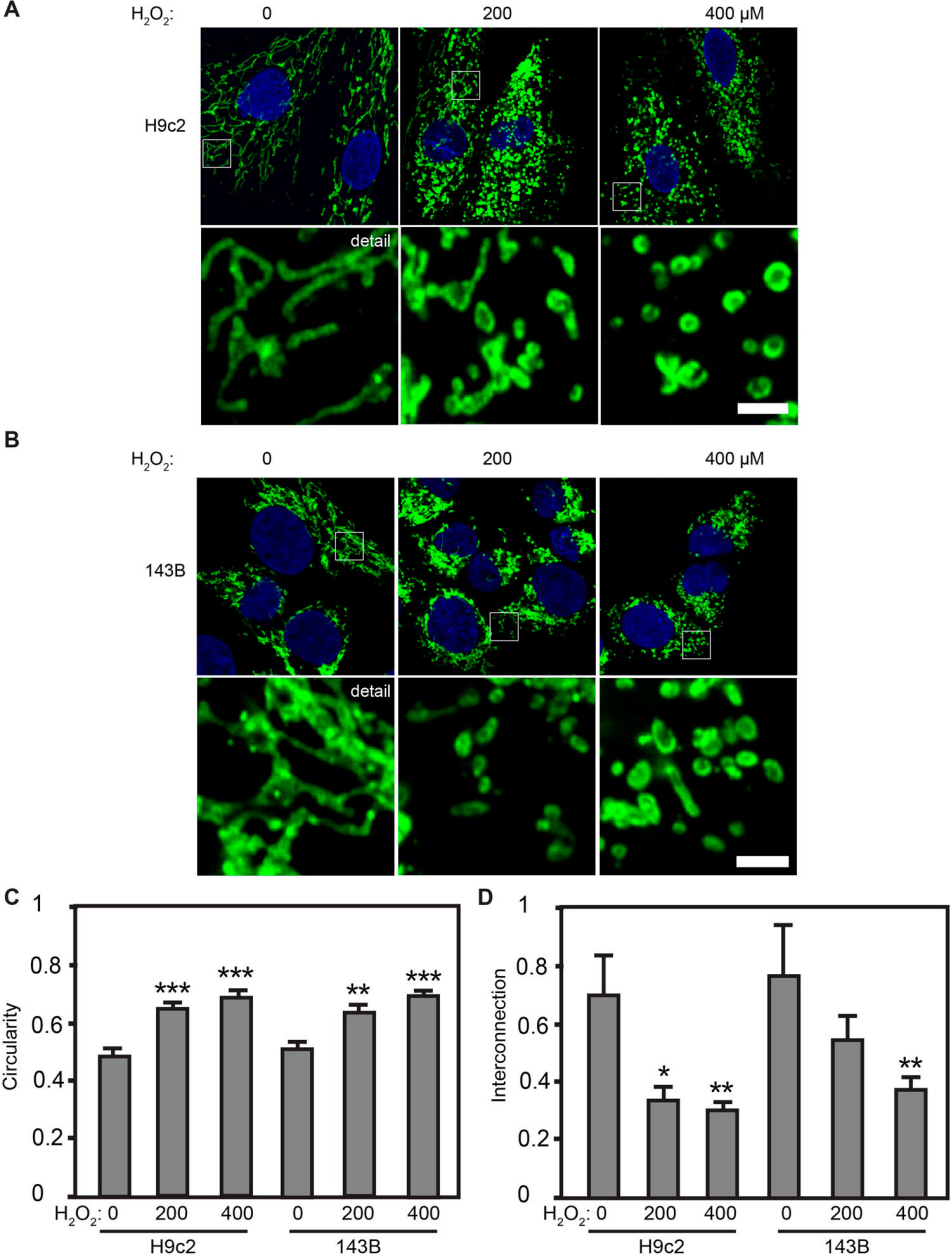
Oxidative stress causes mitochondrial fragmentation. Confocal microscopy of H9c2 (A) and 143B cells (B) immunolabeled for mitochondrial TOM 20 (green), with nuclei stained with DAPI (blue). (C, D) Quantification of mitochondrial morphology parameters for H9c2 and 143B lines. Circularity measures the average value per high-resolution micrograph (see detail images in A, B above for representative examples). Interconnection measures the average area/perimeter/mitochondrial profile per micrograph. *n* = 25, ±SE. *Significant at *P* < .05, **significant at *P* < .01, ***significant at *P* < .0001, one-way ANOVA followed by Tukey *post hoc* test.

To assess mitochondrial morphology, confocal images were used to quantitate both mitochondrial circularity and interconnection via ImageJ, per the method of Dagda et al. [28]. High-resolution images (for examples, see detail images, Figure 1(A,B)) were analyzed using the Mitochondrial Morphology macro with ImageJ (publicly available at http://imagejdocu.tudor.lu/doku.php?id=plugin:morphology:mitochondrial_morphology_macro_plug-in:start). The circularity value describes the average circularity of all mitochondria in the image, where a perfect circle = 1.0. Interconnectivity is calculated from the average area/perimeter for all mitochondria in the image. Reported values were averages for *n* = 25 images of each sample, generated via blinded analysis, and statistically analyzed. All results expressed as mean ± SEM. *P*-values below .05 were statistically significant.

## 3. Results

### 3.1. H_2_O_2_ causes mitochondrial fragmentation in H9c2 and 143B cells

As a mitochondrially localized protein, TOM20 provides a useful marker of mitochondrial organization via immunofluorescence confocal microscopy (Suppl. Fig. 1A). When viewed by anti-TOM20 immunofluorescence, untreated H9c2s showed a mix of both mitochondrial fission and fusion. When challenged with 200 μM H_2_O_2_, however, mitochondria were largely fragmented, while at 400 μM H_2_O_2_, H9c2s showed near-total mitochondrial fragmentation (Figure 1(A)). Similarly, untreated 143Bs showed both interconnected and fragmented mitochondria, while 200 and 400 μM H_2_O_2_ caused extensive mitochondrial fragmentation (Figure 1(B)). ImageJ quantitation confirmed this: when quantitating mitochondrial circularity and interconnection, untreated H9c2s showed a circularity value of 0.488 ± 0.03, compared with significantly increased mitochondrial circularity in H9c2s treated with 200 μM H_2_O_2_ (0.655 ± 0.02) and 400 μM H_2_O_2_ (0.695 ± 0.02) (Figure 1(C)). The increased circularity of mitochondria in H_2_O_2_-treated H9c2s is mirrored by the loss of mitochondrial interconnection (200 μM H_2_O_2_: 0.337 ± 0.04, 400 μM H_2_O_2_: 0.306 ± 0.03) compared with untreated H9c2s (0.699 ± 0.14) (Figure 1(D)). Consistent with this, quantitation of 143Bs showed that H_2_O_2_ elicits mitochondrial fragmentation: untreated 143Bs have a mitochondrial circularity value of 0.512 ± 0.03, while H_2_O_2_ caused a significant increase in circularity at 200 μM (0.643 ± 0.02) and 400 μM H_2_O_2_ (0.695 ± 0.02) (Figure 1(C)). 143B cells treated with 400 μM H_2_O_2_ also showed a significant decrease in mitochondrial interconnection compared with untreated 143Bs (0.376 ± 0.04 versus 0.767 ± 0.12) (Figure 1(D)). To examine whether the use of high glucose DMEM skewed our results, we examined mitochondrial morphology of both H9c2 and 143B cells in high glucose (25 mM) and normoglycemic (5 mM) media. Neither H9c2s nor 143Bs had apparent differences in mitochondrial morphology between the two glucose concentrations (Suppl. Fig. 1B); the statistically equivalent mitochondrial circularity values for each, as determined by Image J, confirmed this (Suppl. Fig. 1C). Taken together, these results show that H_2_O_2_-induced oxidative stress causes fragmentation of the mitochondrial network in both cell lines.

### 3.2. Differential effects of H_2_O_2_ on Δψ_m_ in cardiomyoblast and osteosarcoma cells

**Figure 2.**
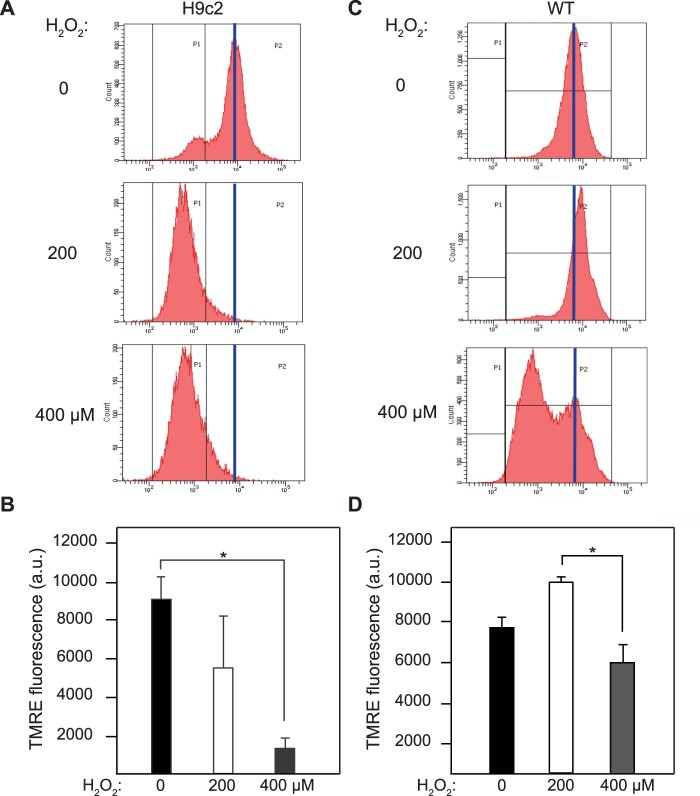
Differential impacts of oxidative stress on Δψ_m._ Δψ_m_ measured by TMRE flow cytometry in H9c2 (A) and 143B (C) lines. Peak TMRE fluorescence represented by vertical blue line. Average TMRE in H9c2 (B) and 143B (D) lines, ±SE. *n* ≥ 3 experiments, 30,000 cells assayed per experiment. *Significant at *P* < .05.

Δψ_m_ was assayed via TMRE flow cytometry, as previously [25,29]. As a Nernstian dye, TMRE accumulates reversibly in mitochondria with an active Δψ_m_. In representative histograms, untreated H9c2s maintain a peak near 10^4^ arbitrary fluorescence units (au) (blue line), while H_2_O_2_-treated H9c2s show dramatic left-shifts in peak TMRE (Figure 2(A)). Untreated H9c2s maintained an average TMRE of 9031 ± 1189 a.u., while H9c2s treated with 400 µM H_2_O_2_ showed a significantly lower TMRE of 1306 ± 532 a.u. (Figure 2(B)). These results contrasted with 143Bs: untreated 143Bs show a single peak, while 143B cells treated with 400 μM H_2_O_2_ show a bimodal distribution, with a second peak showing a distinct left-shift (Figure 2(C)). As average TMRE was calculated for all events (no gating), no significant difference was found between the untreated and H_2_O_2_-treated 143Bs (Figure 2(D))*.* The bimodal distribution found in H_2_O_2_-treated 143B cells indicates that two subpopulations exist, possibly reflecting differences in cell cycle requirements for mitochondrial bioenergetics; however, this remains to be explored in detail. Collectively, these results demonstrate that identical oxidative stresses may have distinct impacts on different cell types, depending on their specific metabolic and bioenergetics demands.

### 3.3. DRP1 levels are not increased in response to H_2_O_2_ challenge in either H9c2 or 143B cell lines

**Figure 3.**
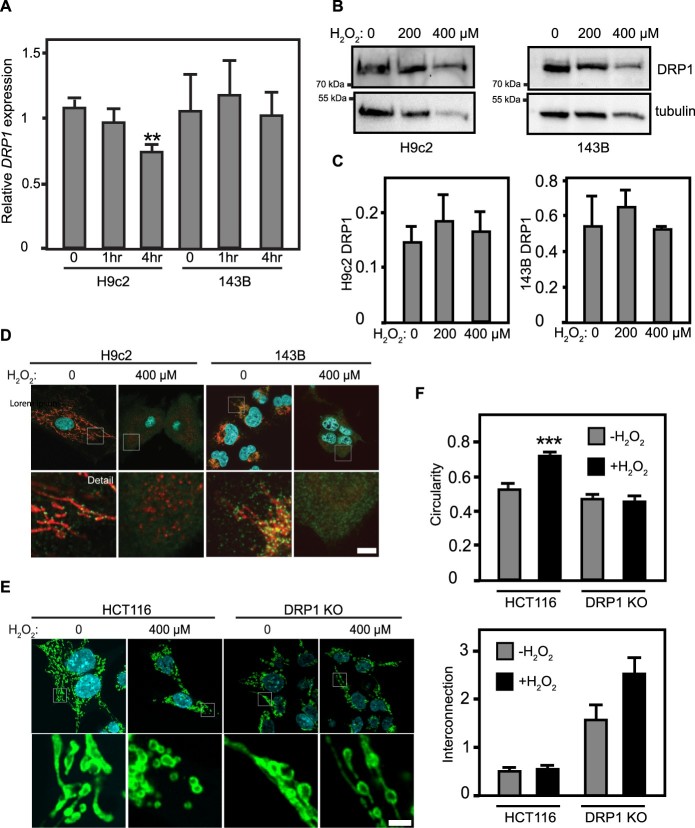
DRP1 expression in cells is not affected by oxidative stress. (A) qRT-qPCR of *DRP1* mRNAs in H9c2s and 143Bs at 0, 1, and 4 h. Treatment with 400 μM H_2_O_2_, ±SE. **statistically significant, *P* < .01. (B) Anti-DRP1 Western blotting of H9c2 cells, without and with 1 h H_2_O_2_. DRP1 and α-tubulin labeled as indicated. (C) ImageJ quantification of DRP1 Westerns. No significant difference observed. ±SE. (D) Microscopy of H9c2 and 143B cells immunolabeled for DRP1 (green) and Mitotracker (red), nuclei labeled with DAPI (cyan). Size bar = 2 μm. (E) Confocal microscopy of HCT116 control and *DRP1* KO cell lines immunolabeled for TOM20 (green) and DAPI (cyan). Size bar = 2 μm. (F) Quantification of mitochondrial morphology parameters for HCT116 control and *DRP1* KO cell lines immunolabeled in (E). ***significant at *P* < .0001, one-way ANOVA followed by Tukey *post hoc* test.

The fragmentation observed in Figure 1 clearly demonstrates a disruption of mitochondrial dynamics. We next sought to determine the mechanism behind the observed mitochondrial fragmentation, hypothesizing that increased levels of DRP1 could be causing fragmentation through increased mitochondrial fission. To explore this, we examined the levels of DRP1 transcripts in both cell lines after 1 or 4 h of treatment with 400 μM H_2_O_2_. Strikingly, neither H9c2s or 143Bs showed a significant increase in *DRP1* mRNA in response to H_2_O_2_ treatment: relative *DRP1* expression (normalized to actin) of 143B cells at 1 h (1.17 ± 0.3) and 4 h (1.01 ± 0.2) were statistically equivalent to untreated 143Bs (1.05 ± 0.3), while H9c2 cells at 4 h (0.739 ± .06) were *decreased* relative to untreated H9c2s (1.08 ± .07) (Figure 3(A)). We next examined whether DRP1 protein levels changed in response to H_2_O_2_. However, Western blotting did not show increased DRP1 in H9c2s or 143Bs treated with for 1 h at 200 or 400 μM H_2_O_2_ (Figure 3(B)). Quantification of DRP1 Western blot signal confirms this: neither H9c2 nor 143B cells showed any significant increase in DRP1 signal (normalized to tubulin loading control) when challenged with H_2_O_2_ (Figure 3(C)).

Alternately, existing DRP1 could be more strongly recruited to H9c2 mitochondria. Anti-DRP1 immunofluorescence of untreated H9c2s shows that DRP1 is concentrated at discrete foci along the mitochondria. Intriguingly, H_2_O_2_-treated cells show a diffuse distribution, with a more pervasive cytosolic distribution (Figure 3(D)). Comparison side-by-side with DRP1 knockout cells (not shown) indicates that this diffuse DRP1 signal in H_2_O_2_-treated H9c2s reflects a genuine redistribution of DRP1. 143B cells yielded similar results: untreated 143B cells revealed DRP1 at discrete foci along the mitochondria, while H_2_O_2_-treated cells show a more diffuse overall distribution within the cell (Figure 3(D)). Though intriguing, these results do not demonstrate increased DRP1 foci at mitochondria in response to H_2_O_2_.

To directly test DRP1’s requirement in H_2_O_2_-mediated mitochondrial fragmentation in a genetically clean model, we next examined HCT116 colorectal carcinoma cells with (control) or lacking DRP1 (DRP1 KO). These cells allow a direct interrogation of DRP1’s role, as a complementary approach to the experiments above in H9c2 and 143B cell lines. While HCT116s showed the expected balance of mitochondrial fission and fusion, H_2_O_2_-challenged HCT116s displayed near-total mitochondrial fragmentation. Conversely, DRP1 KO cells show extensive mitochondrial interconnection, consistent with a lack of DRP1-mediated fission. Strikingly, H_2_O_2_-treated DRP1 KO cells strongly retain mitochondrial interconnection, in contrast to the massive fragmentation shown in H_2_O_2_-treated control cells (Figure 3(E)). ImageJ analysis of mitochondrial circularity confirmed this, showing that HCT116 control cells significantly increased mitochondrial circularity when challenged with H_2_O_2_, while DRP1 KO cells do not (Figure 3(F)). Thus, while DRP1 levels and mitochondrial recruitment do not appear to increase in response to H_2_O_2_ treatment in H9c2 and 143B cell lines, it remains possible that DRP1 plays a contributing role in oxidatively induced mitochondrial fragmentation. However, as DRP1-mediated fission is mechanistically opposed by OPA1-mediated mitochondrial fusion, we next examined OPA1 as a potential target of H_2_O_2_-mediated oxidative stress.

### 3.4. H_2_O_2_ challenge causes L-OPA1 cleavage in both H9c2 cardiomyoblasts and 143B osteosarcoma cells

**Figure 4.**
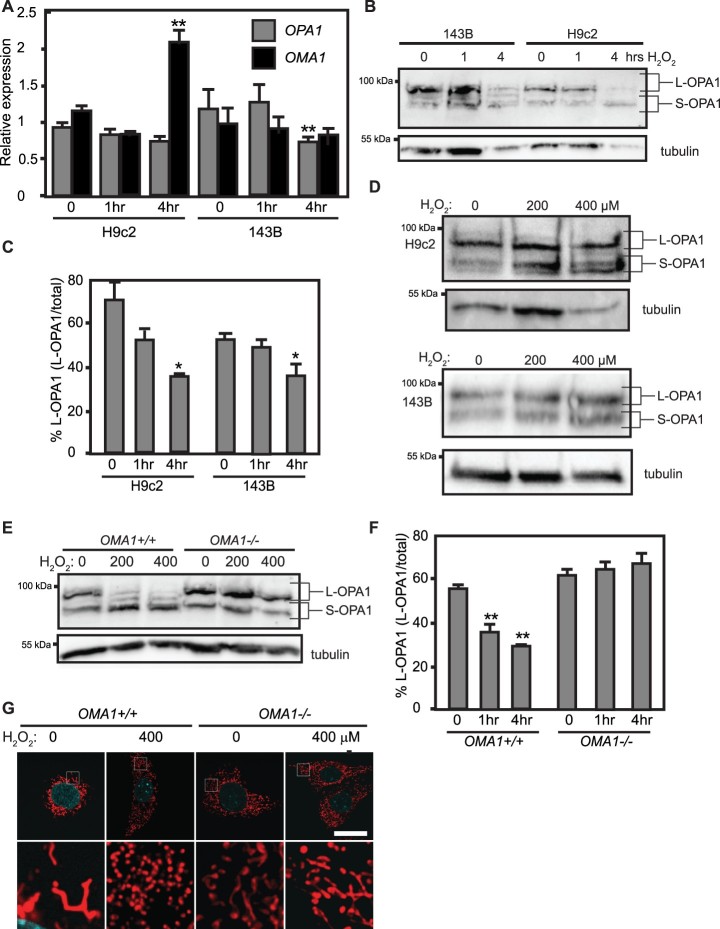
Impacts of oxidative insults on OPA1 and OMA1. (A) qRT-PCR of *OPA1* and *OMA1* mRNAs in H9c2s 143Bs at 0, 1, and 4 h. treatment with 400 μM H_2_O_2_, ±SE. **statistically significant from corresponding untreated sample, *P* < .01. (B) Anti-OPA1 Western blotting of H9c2s and 143Bs at 0, 1, and 4 h. treatment with 400 μM H_2_O_2_. L- and S-OPA1 isoforms labeled as indicated. (C) ImageJ quantification of L-OPA1 levels in (B), *n* = 3. *Significant at *P* < .05, one-way ANOVA followed by Tukey *post hoc* test. (D) Anti-OPA1 blotting of cells treated with H_2_O_2_ for 1 h at indicated concentrations. (E) Anti-OPA1 blotting of *OMA1+/+* and *−/−* cells treated with H_2_O_2_ for 4 h at indicated concentrations. (F) ImageJ quantification of L-OPA1 levels in (E), *n* = 3. **statistically significant from corresponding untreated sample, *P* < .01., one-way ANOVA followed by Tukey *post hoc* test. (G) Confocal microscopy of *OMA1+/+* and *OMA1−/−* MEFs without or with H_2_O_2_. *n* = 3 experiments. Size bar = 10 μm.

To examine the involvement of OPA1 and its Δψ_m_-sensitive protease OMA1 in H_2_O_2_-mediated mitochondrial fragmentation, we examined the levels of *OPA1* and *OMA1* mRNA transcripts after 1 and 4 h of H_2_O_2_ challenge. H9c2 cardiomyoblast *OPA1* expression (normalized to actin) did not show a significant change in response to H_2_O_2_; 143B cells showed a decrease in *OPA1* expression after 4 h of H_2_O_2_ (Figure 4(A)). 143B cells did not show any change in *OMA1* levels in response to H_2_O_2_. Intriguingly, H9c2 cells showed a robust 2-fold increase in *OMA1* expression after 4 h of H_2_O_2_ treatment (Figure 4(A)). To examine whether the fusion-active L-OPA1 isoforms were affected by H_2_O_2_ challenge, we examined the OPA1 isoforms present in control and H_2_O_2_-treated cell lysates. OPA1 exists as five different isoforms (a, b, c, d, and e). Δψ_m_-sensitive inner membrane fusion is mediated by the a and b long isoforms (L-OPA1), while the short c, d, and e isoforms (S-OPA1) are fusion-inactive [7]. Constitutive proteases including YME1L result in steady-state production of S-OPA1 isoforms [30]. Upon loss of Δψ_m_, L-OPA1 isoforms are cleaved by OMA1 [8,9]. OPA1 blotting of untreated H9c2 and 143B lysates revealed the expected balance of both L-OPA1 and S-OPA1. In response to 4 h of H_2_O_2_ treatment, however, L-OPA1 bands were lost: while untreated 143B and H9c2 cells showed a robust major band in the L-OPA1 isoforms, this was lost in the 4 h H_2_O_2_ lysates (Figure 4(B)), indicating that L-OPA1 was cleaved to S-OPA1 in response to H_2_O_2_. ImageJ quantification confirmed this: L-OPA1 levels in untreated H9c2 (71 ± 11%) and 143B (52 ± 2%) lysates were significantly greater than in 4 h H_2_O_2_-treated lysates for both H9c2 (36 ± 1.4%) and 143B (36 ± 5%) cell lines (Figure 4(C)). These findings confirm that fusion-active L-OPA1 is lost in both cell lines in response to H_2_O_2_ challenge. We next examined lysates of both cell lines treated for 1 h with 200 and 400 μM H_2_O_2_. In H9c2 cells, H_2_O_2_ treatment appears to cause an accumulation of S-OPA1 isoforms relative to untreated controls. 143B cells showed no appreciable differences (Figure 4(D)); these observations were confirmed by ImageJ quantification (not shown). Taken together, these results indicate that while overall expression of *OPA1* does not increase in response to H_2_O_2_ challenge, both H9c2 and 143B cell lines show loss of L-OPA1 isoforms in response to H_2_O_2_ treatment. As OMA1 is the major inducible OPA1 protease [8,9], we next examined whether OMA1 is required for H_2_O_2_-mediated L-OPA1 cleavage and mitochondrial fragmentation.

### 3.5. Cells lacking OMA1 are protected against H_2_O_2_-mediated OPA1 cleavage and mitochondrial fragmentation

The mitochondrial fragmentation (Figure 1) and loss of L-OPA1 (Figure 4(B,C)), as well as the robust upregulation of OMA1 in H_2_O_2_-treated H9c2s (Figure 4(A)), strongly suggest that OMA1 plays a major role in H_2_O_2_-mediated disruption of mitochondrial dynamics. To directly test whether OMA1 is required, we examined mouse embryonic fibroblast (MEFs) containing (*OMA1+/+*) or lacking (*OMA1−/−*) the *OMA1* gene. Western blotting of control *OMA1+/+* cells revealed the expected balance of L-OPA1 and S-OPA1 isoforms, with a robust band for the L-OPA1 b isoform. In response to treatment with 200 or 400 μM H_2_O_2,_ however, L-OPA1 was visibly lost compared to untreated controls. Strikingly, however, *OMA1−/−* cells showed robust L-OPA1 in control and H_2_O_2_-treated lysates (Figure 4(E)). ImageJ quantification confirmed that *OMA1+/+* cells showed highly significant decreases in L-OPA1 in response to H_2_O_2,_ while *OMA1−/−* cells showed no difference in L-OPA1 levels between untreated controls and H_2_O_2_-treated samples (Figure 4(F)). Under confocal microscopy, untreated *OMA1+/+* MEFs had predominantly interconnected mitochondria, as visualized by MitoTracker. When challenged with H_2_O_2_, however, control MEFs showed near-total mitochondrial fragmentation (Figure 4(G)). Conversely, *OMA1−/−* MEFs challenged with 200 or 400 μM H_2_O_2_ maintained readily apparent mitochondrial interconnection (Figure 4(G)). These experiments show that cells lacking OMA1 do not display the H_2_O_2_-mediated mitochondrial fragmentation and L-OPA1 cleavage effects observed above. Taken together, these findings strongly suggest that OMA1 is activated in response to oxidative insult in cultured cells.

## 4. Discussion

Here, we show that H_2_O_2_-mediated oxidative stress disrupts mitochondrial dynamics in both H9c2 cardiomyoblasts and 143B osteosarcomas, causing fragmentation of the mitochondrial network and cleavage of fusion-active L-OPA1 isoforms. Moreover, cells lacking the OMA1 metalloprotease are protected from these impacts, retaining L-OPA1 and mitochondrial interconnection under H_2_O_2_ challenge. These findings support a role for OMA1 as a key sensor of oxidative impacts on mitochondrial dynamics, and suggest that OPA1-mediated mitochondrial fusion may be a critical early indicator of oxidative stress for subsequent cellular stress signaling.

Despite their different backgrounds, H_2_O_2_ causes extensive mitochondrial fragmentation in both 143B osteosarcomas and H9c2 cardiomyoblasts, with striking effects on both mitochondrial circularity and interconnection parameters (Figure 1). This rapid fragmentation of the mitochondrial network causes significant loss of Δψ_m_ in H9c2s, but not 143Bs (Figure 2). Mechanistically, the mitochondrial fragmentation observed does not appear to require DRP1: neither H9c2s nor 143Bs showed increased DRP1 expression. Despite this, DRP1 knockout cells were insensitive to H_2_O_2_, indicating that DRP1 may still play a role in oxidative fragmentation of mitochondria. Strikingly, however, *both* cell lines showed loss of the long, fusion-mediating, L-OPA1 isoforms in response to H_2_O_2_ challenge, with H9c2s showing a concomitant twofold increase in *OMA1* transcripts (Figure 4). These findings strongly implicate OPA1-mediated mitochondrial fusion as a mechanistic target of oxidative stress, further strengthened by our findings that cells lacking the OMA1 protease are insensitive to H_2_O_2_-mediated mitochondrial fragmentation and L-OPA1 cleavage.

While OMA1 has been extensively characterized for its ability to cleave L-OPA1 in response to uncoupling agents such as CCCP [8,9], our results are consistent with a broader role for OMA1 and its cleavage of OPA1 as a key mitochondrial stress response mechanism [31]. Future work is needed to explore the functional determinants of OMA1 activation: an N-terminal domain appears to be responsible for CCCP-mediated activation of OMA1 [32], providing an insight motivating further characterization of OMA1 proteolytic determinants, as well as the interacting proteins that help dictate functional responses. *In vivo,* excessive OPA1 cleavage causes dilated cardiomyopathy and heart failure [33], while loss of OPA1 also causes inflammation in muscle [12] through activation of UPR and inflammatory signaling [13]. As such, OMA1’s role in controlling mitochondrial structure/function homeostasis and downstream signaling outputs is a prime candidate for further exploration as a checkpoint in cellular stress response.

## Supplementary Material

Supplemental Material
