# Supplementary figures and images for: Oxidative insults disrupt OPA1-mediated mitochondrial dynamics in cultured mammalian cells
Source: Redox Rep. 2018 Jul 1;23(1):160–7. doi: 10.1080/13510002.2018.1492766 (PMC6272060; doi:10.1080/13510002.2018.1492766)

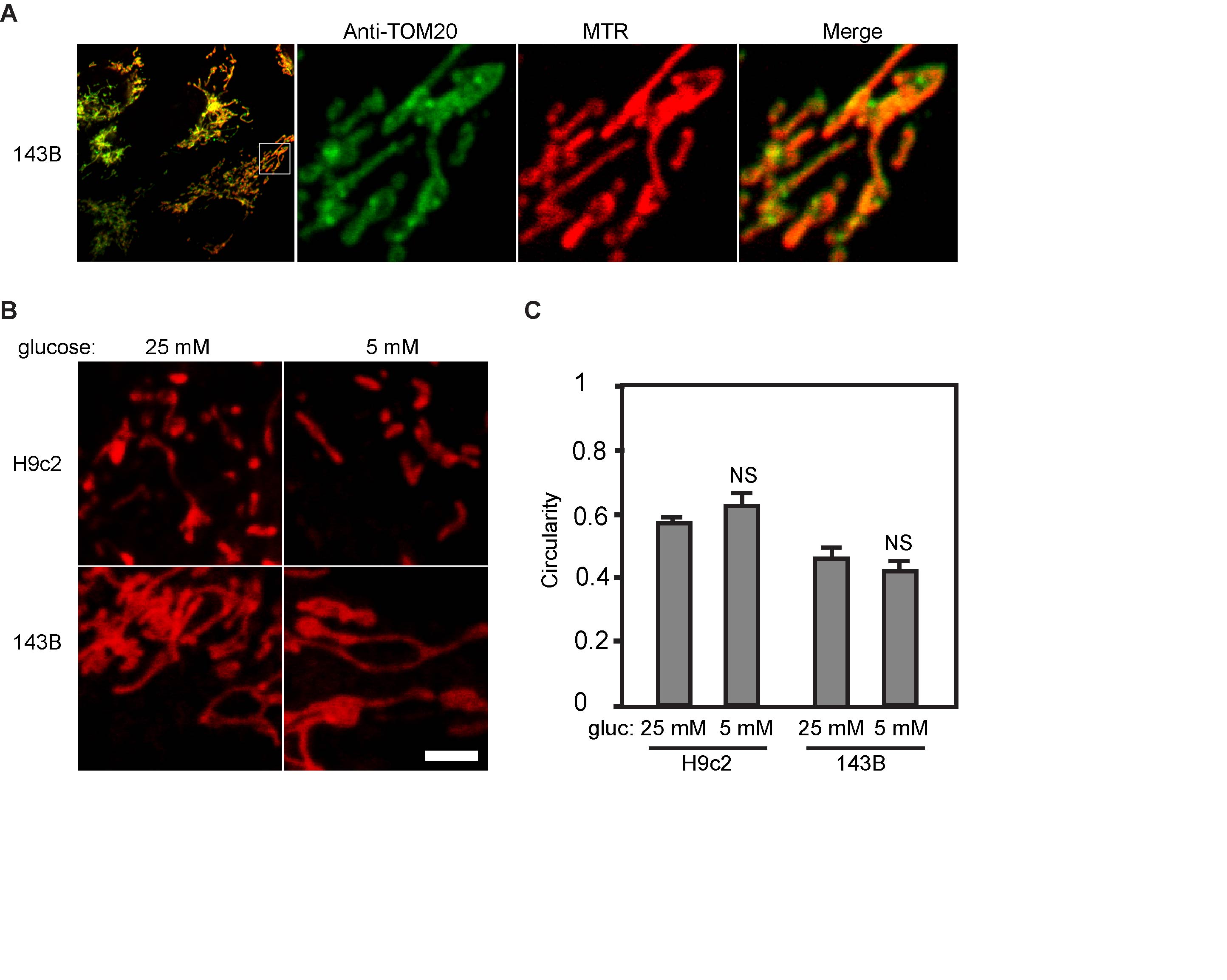

Supplement: Supplemental Material [file YRER_A_1492766_SM8859.jpg]
